# Clinical Parameters Associated with Achieving Negative Fluid Balance in Critically Ill Patients: A Retrospective Cohort Study

**DOI:** 10.3390/jcm15020764

**Published:** 2026-01-17

**Authors:** Dekel Stavi, Amir Gal Oz, Nimrod Adi, Roy Rafael Dayan, Yoel Angel, Andrey Nevo, Nardeen Khoury, Itay Moshkovits, Yael Lichter, Ron Wald, Noam Goder

**Affiliations:** 1 Division of Anaesthesia, Pain Management and Intensive Care, Tel Aviv Sourasky Medical Center, Weizmann 6, Tel Aviv 6423919, Israel; amirgo@tlvmc.gov.il (A.G.O.); nimroda@tlvmc.gov.il (N.A.); roydayan@tlvmc.gov.il (R.R.D.); yoela@tlvmc.gov.il (Y.A.); andreyne@tlvmc.gov.il (A.N.); nardeenk@tlvmc.gov.il (N.K.); itaymo@tlvmc.gov.il (I.M.); noam.goder@mail.utoronto.ca (N.G.); 2 Gray Faculty of Medical and Health Science, Tel Aviv University, Tel Aviv 6997801, Israel; 3 Critical Care Department, University College London Hospital NHS Foundation Trust, 235 Euston Rd, London NW12BU, UK; yael.lichter@nhs.net; 4 Division of Nephrology, St. Michael’s Hospital and the University of Toronto, 36 Queen St, Toronto, ON M5B 1W8, Canada; ron.wald@unityhealth.to; 5 Li Ka Shing Knowledge Institute of St. Michael’s Hospital, 209 Victoria St, Toronto, ON M5B 1T8, Canada; 6 Division of Surgery, Tel Aviv Sourasky Medical Center, Weizmann 6, Tel Aviv 6423919, Israel

**Keywords:** critical care, fluid management, fluid balance, fluid accumulation, de-resuscitation

## Abstract

**Background/Objectives:** Fluid overload in critically ill patients is linked to adverse outcomes. While resuscitation strategies are well established, guidance for the de-resuscitation phase remains limited. This study aimed to identify clinical factors associated with diuretic response and achieving negative fluid balance (FB) in critically ill patients. **Methods:** We conducted a single-center, retrospective cohort study of ICU patients who received intravenous furosemide between 2017 and 2023. A CHAID (Chi-square Automatic Interaction Detector) decision tree identified clinical variables associated with fluid removal after the first dose, and a mixed-effects model analyzed repeated measurements. **Results:** The cohort comprised 1764 patients over 6632 ICU days. Mean arterial pressure (MAP) was the strongest predictor of negative FB. MAP ≤ 75 mmHg yielded minimal negative FB (−33 ± 1054 mL/24 h); MAP 75–90 mmHg yielded intermediate negative FB (−467 ± 1140 mL/24 h); and MAP > 90 mmHg produced the greatest negative FB (−899 ± 1415 mL/24 h; *p* < 0.001). Secondary associations varied by MAP: creatinine at low MAP, blood urea nitrogen at mid-range MAP, and SOFA score at high MAP, all inversely related to negative FB. In mixed-effects analyses, each 1 mmHg MAP increase was associated with 23.3 mL greater fluid removal (*p* < 0.001). Independent factors linked to reduced negative FB included vasopressor use (noradrenaline), elevated creatinine, and higher SOFA scores. **Conclusions:** In this cohort, MAP was significantly associated with the likelihood of achieving a negative fluid balance during de-resuscitation. Conversely, vasopressor use, renal dysfunction, and higher illness severity were linked to reduced diuretic responsiveness. These findings support individualized de-resuscitation strategies.

## 1. Introduction

Fluid accumulation is common in critically ill patients, particularly in the early stages of treatment when fluid administration is often necessary [1,2,3]. Capillary leakage and the shift of fluid from the intravascular to the interstitial compartment are driven by an interplay of endothelial barrier disruption, glycocalyx shedding, and reduced plasma oncotic pressure. The resulting interstitial edema impairs the diffusion of oxygen and nutrients, impedes lymphatic return, and alters cell–cell interactions [4,5,6,7,8]. This volume overload and subsequent edema are associated with adverse consequences such as prolonged mechanical ventilation, various organ complications, impaired gas exchange, increased intra-abdominal pressure and delayed wound healing [4,9,10,11,12]. While the goals and methods of fluid management during the resuscitative phase are extensively discussed, uncertainty regarding methods of fluid removal during the de-resuscitation phase remains [3,4,10]. After hemodynamic stabilization is achieved, vasoactive therapy is typically de-escalated, and efforts are made to remove excess fluids by aiming for a negative fluid balance (FB) [13,14,15,16,17]. This goal is usually achieved by mitigating fluid intake and actively removing of fluids either by pharmacological therapy or by mechanical fluid removal techniques [11]. The initial steps taken for active fluid removal usually involve pharmacological therapy, mostly with the use of loop diuretics. However, even when targeting a negative FB, achieving this goal remains clinically challenging and unpredictable. Success is often hampered by factors such as impaired kidney function, hemodynamic instability, profound hypoalbuminemia, and inter-patient variability in the duration of natriuretic effects [5,11,14,18].

This study aims to retrospectively identify and analyze factors associated with the successful attainment of a predefined negative FB through pharmacological therapy in critically ill patients.

## 2. Materials and Methods

Study design and data source: We conducted a retrospective cohort study, in the General Intensive Care Unit (ICU) at Tel Aviv Medical Center, a tertiary medical center in Israel, between January 2017 and December 2023. Inclusion criteria encompassed ICU days on which patients had documented administration of intra venous furosemide. Exclusion criteria included ICU days with missing urine output documentation, daily fluid intake exceeding 2500 mL, or a daily furosemide dose greater than 500 mg. In addition, patients who required dialysis or continuous kidney replacement therapy at any time during their ICU stay were excluded. Data collected for each patient day included fluid intake and output. Daily FB was then calculated and analyzed in relation to various associated variables.

Demographic variables included age (years), sex, length of stay (LOS) in the ICU before furosemide was given, and the Sequential Organ Failure Assessment (SOFA) score on the day furosemide was administered. The laboratory data included results from routine daily blood test results collected each morning around 6:00 a.m., measuring plasma levels of creatinine (mg/dL), albumin (g/dL), sodium (mmol/L), blood urea nitrogen (BUN, mg/dL), hemoglobin (Hb, g/dL), and bicarbonate (mmol/L). Hemodynamic monitoring was conducted from 06:00 a.m. on the day of furosemide administration to 06:00 AM the following day, recording the mean arterial pressure (MAP, mmHg), mean daily heart rate (HR, bpm), and, for mechanically ventilated patients, the mean positive end-expiratory pressure (PEEP, cmH_2_O). Mean arterial pressure (MAP) was obtained primarily from an indwelling arterial catheter; validated hourly values were automatically captured in the electronic medical record. When an arterial line was not available or an hourly value was missing, non-invasive oscillometric cuff measurements were used. Hourly MAP values recorded during the 24 h study window (06:00 on the day of furosemide administration to 06:00 the following day) were averaged to generate a single mean daily MAP for each patient-day. Additionally, the peak temperature within 24 h of diuretic administration was documented. Recorded interventions included noradrenaline support and the daily furosemide dose (mg). Assessed past medical history variables included hypertension, diabetes mellitus, congestive heart failure, ischemic heart disease, cirrhosis, metastatic cancer, hematologic malignancy, HIV, and history of major surgery (including aortic and cardiac procedures), as well as whether the ICU admission was due to trauma, sepsis, or a postoperative condition.

Ethical approval for this study (IRB No. TLV-0061-24) was obtained from the Sourasky Medical Center Ethics Committee, on 15 February 2024. The requirement for informed consent was waived as all data were fully anonymized prior to access and analysis.

Statistical analysis: Continuous variables are presented as means with standard deviations (SD) or medians with interquartile ranges (IQR), as appropriate. Categorical variables are reported as frequencies and percentages. To explore determinants of achieving a negative FB, we employed the CHAID (Chi-squared Automatic Interaction Detector) decision tree algorithm, which allowed us to construct a hierarchical decision tree for identifying and visualizing the most significant variables influencing FB in patients treated with furosemide [19,20]. Since the CHAID model does not handle repeated measurements, we restricted its use to data from the first day of furosemide administration (one record per patient) to observe initial variable importance and correlations with negative balance outcomes. In addition, a mixed linear model was used to account for repeated measurements across multiple ICU days for individual patients, capturing within-subject variability over time. This model allowed us to include all available data while addressing the clustering of repeated measurements. Estimates in the mixed models are presented with means and 95% confidence interval (CI).

Further analysis included paired t-tests for matched comparisons of creatinine, BUN, sodium, and albumin levels measured on the mornings before and after furosemide administration, aiming to isolate the impact of furosemide on kidney function from individual patient variability. Statistical analyses were conducted with SPSS (IBM, version 29).

## 3. Results

**Figure 1:**
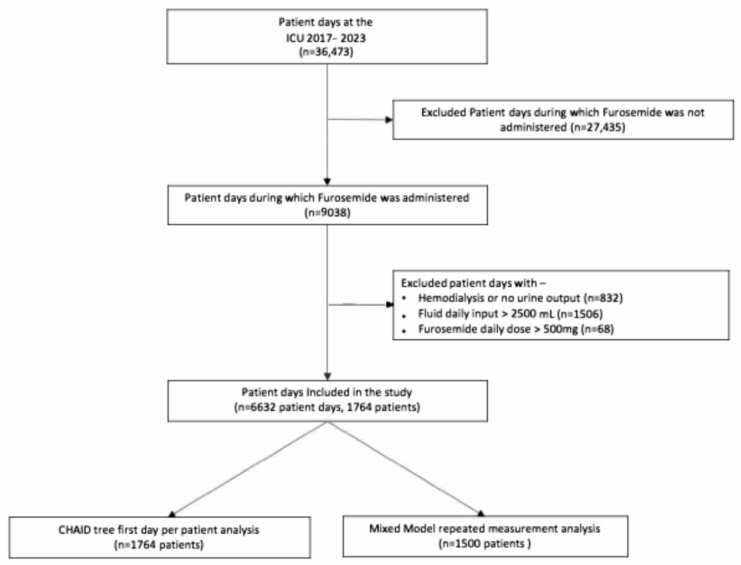
Patient ICU days inclusion for the study.

A total of 36,473 ICU patient-days were screened for furosemide administration. After excluding patients as outlined in Figure 1, the study analyzed 6632 patient-days, involving 1764 patients who received furosemide. Demographic details and daily collected data are summarized in Table 1.

### 3.1. Factors Associated with Fluid Balance Following the First Furosemide Administration

**Figure 2:**
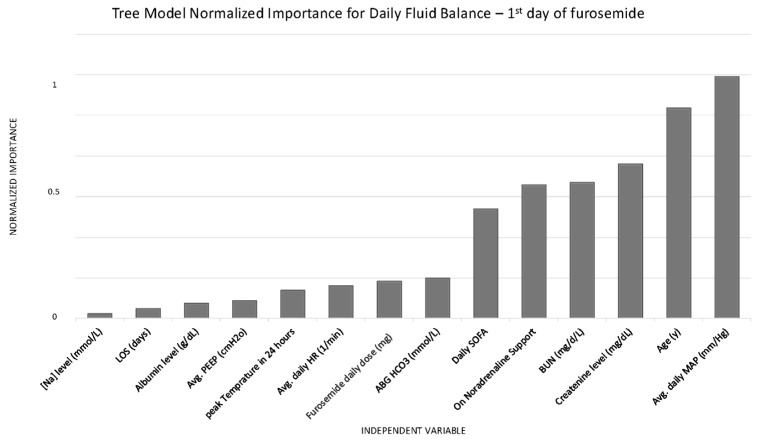
Decision tree model showing normalized importance of variables associated with daily fluid balance on the first day of furosemide administration. LOS—length of stay, Avg.—average, ABG—arterial blood gas, SOFA—sequential organ failure assessment, BUN—blood urea nitrogen, MAP—mean arterial pressure.

**Figure 3:**
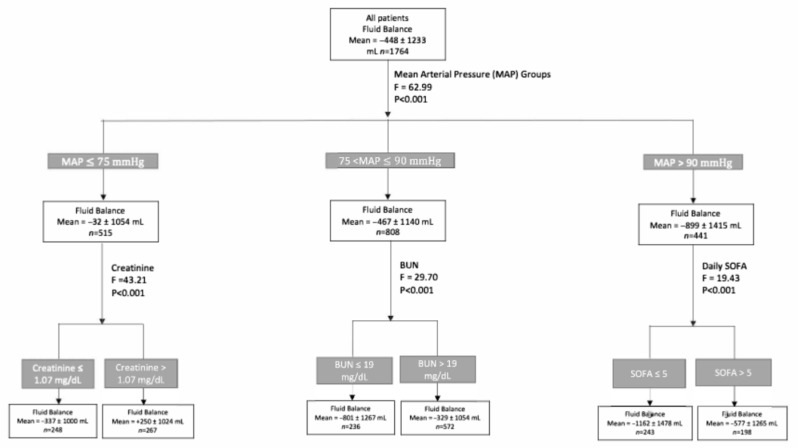
Daily fluid balance following the 1st day of furosemide usage by MAP. BUN—blood urea nitrogen, SOFA—sequential organ failure assessment.

A CHAID decision tree analysis was applied to examine daily FB on the first day of treatment, using normalized importance to identify key associated variables (Figure 2). The analysis identified mean daily MAP as the variable most strongly associated with FB following furosemide administration. MAP was stratified into three categories based on model- suggested thresholds: ≤75 mmHg, 75–90 mmHg, and >90 mmHg (Figure 3). FB differed significantly across these MAP categories: patients with a mean daily MAP ≤ 75 mmHg (n = 515) had a mean FB of −33 ± 1054 mL/24 h, those with MAP between 75 and 90 mmHg (n = 808) showed a balance of −467 ± 1140 mL/24 h, and patients with MAP > 90 mmHg (n = 441) exhibited the most negative fluid balance at −899 ± 1415 mL/24 h (F = 62.99, *p* < 0.001).

Further analysis identified distinct clinical variables associated with FB within each MAP category. Among patients with a MAP ≤ 75 mmHg, serum creatinine emerged as the most relevant associated factor: those with creatinine < 1.07 mg/dL had a mean FB of −337 ± 1000 mL/24 h, compared to +250 ± 1024 mL/24 h in patients with creatinine ≥ 1.07 mg/dL (F = 43.21, *p* < 0.001). In the 75–90 mmHg MAP group, blood urea nitrogen (BUN) was significantly associated with FB, with patients exhibiting BUN < 19 mg/dL demonstrating a more negative balance (−801 ± 1267 mL/24 h) than those with BUN ≥ 19 mg/dL (−329 ± 1054 mL/24 h; F = 29.70, *p* < 0.001). Among patients with MAP > 90 mmHg, the SOFA score was the primary associated variable: patients with SOFA scores ≤ 5 had a mean FB of −1162 ± 1478 mL/24 h, whereas those with scores > 5 had a less negative balance of −577 ± 1265 mL/24 h (F = 19.43, *p* < 0.001).

### 3.2. Mixed Model Analysis Encompassing All Measurements

In a mixed model analysis of 1500 ICU patients with repeated measurements, several clinical factors were significantly associated with FB. Higher MAP and greater cumulative furosemide dose were associated with more negative FB, while noradrenaline use correlated with more positive balances. Impaired diuretic response and negative FB achievement was further linked to elevated creatinine, older age, higher SOFA scores, longer ICU stay prior to furosemide administration, and lower bicarbonate levels. In contrast, comorbidities such as hypertension, diabetes, heart failure, ischemic heart disease, cirrhosis, and metastatic cancer showed no significant association. Specifically, each 1 mmHg increase in MAP was associated with a −23.3 mL change in FB (*p* < 0.001), while noradrenaline use was associated with +328 mL (*p* < 0.001). Each additional milligram of furosemide resulted in −3.4 mL (*p* < 0.001). Higher creatinine (+174 mL per mg/dL), age (+9.65 mL per year), SOFA score (+28.24 mL per point), and ICU length of stay prior to furosemide (+4.06 mL/24 h) were all independently associated with more positive fluid balances (all *p* < 0.005). Full model results are presented in Table 2.

### 3.3. Kidney Function and Laboratory Changes Following Initial Furosemide Administration

Among ICU patients receiving furosemide for the first time, modest laboratory changes were observed within 24 h. Creatinine increased slightly from 1.33 ± 1.19 to 1.41 ± 1.35 mg/dL (n = 1335, *p* < 0.001), and BUN rose from 34.90 ± 23.88 to 38.45 ± 26.56 mg/dL (n = 1362, *p* < 0.001). Sodium levels showed a minor increase from 140.82 ± 5.57 to 141.58 ± 5.54 mmol/L (n = 1367, *p* < 0.001), while albumin levels decreased slightly from 28.45 ± 5.21 to 28.28 ± 4.89 g/dL (n = 1242, *p* = 0.014). These changes were statistically significant but of limited clinical relevance.

### 3.4. The Association Between Achieved Fluid Balance and Vasopressor Support on Kidney Function

To assess the association between achieved FB and kidney function and laboratory parameters, patients were stratified for exploratory analyses based on whether their daily FB on the first day of furosemide administration was above or below the cohort mean (−448 mL/24 h).

Among patients with a less negative FB (>−448 mL/24 h), there was a statistically significant upward trend in creatinine levels over 24 h, rising from 1.51 ± 1.29 mg/dL to 1.65 ± 1.49 mg/dL (n = 779, *p* < 0.001). Similarly, BUN levels showed an increase from 38.44 ± 25.58 mg/dL to 43.60 ± 28.55 mg/dL (n = 796, *p* < 0.001). Conversely, changes in albumin and sodium levels were minimal: albumin decreased from 28.47 ± 5.20 g/dL to 27.88 ± 4.84 g/dL (n = 708, *p* < 0.001), while sodium increased from 140.54 ± 5.60 mmol/L to 141.29 ± 5.56 mmol/L (n = 800, *p* < 0.001).

In contrast, patients with a more negative FB (<–448 mL/24 h) exhibited only minor fluctuations in laboratory parameters, all of which are likely to be of limited clinical relevance. Creatinine levels showed a negligible change, decreasing slightly from 1.08 ± 0.98 mg/dL to 1.07 ± 1.04 mg/dL (n = 556, *p* = 0.521). Albumin levels increased modestly, from 28.42 ± 5.23 g/dL to 28.81 ± 4.91 g/dL (n = 534, *p* < 0.001), and sodium rose marginally from 141.22 ± 5.50 mmol/L to 141.99 ± 5.49 mmol/L (n = 567, *p* < 0.001). BUN levels also increased slightly, from 29.92 ± 20.26 mg/dL to 31.20 ± 21.50 mg/dL (n = 566, *p* < 0.001), though the clinical impact of this change appears minimal.

In a subgroup analysis of patients on noradrenaline support receiving furosemide, creatinine levels increased from 1.54 ± 1.17 mg/dL to 1.68 ± 1.36 mg/dL (n = 315, *p* < 0.001). BUN levels also rose from 41.68 ± 27.58 mg/dL to 47.63 ± 31.12 mg/dL (n = 324, *p* < 0.001). Both albumin and sodium levels showed modest changes, with albumin decreasing slightly from 26.94 ± 4.83 g/dL to 26.45 ± 4.55 g/dL (n = 291, *p* = 0.001), and sodium increasing from 141.27 ± 5.90 mmol/L to 142.37 ± 5.87 mmol/L (n = 325, *p* < 0.001).

## 4. Discussion

This study yielded several key findings. (1) MAP was most strongly associated with achieving a negative FB following furosemide administration. (2) Secondary associations varied by MAP category: creatinine levels were significant at low MAP, BUN at mid-range MAP, and SOFA scores at high MAP. (3) Mixed model analysis revealed that higher MAP and greater cumulative furosemide doses were significantly associated with enhanced fluid removal, while noradrenaline support, elevated creatinine levels, and higher SOFA scores were associated with reduced diuretic efficacy. (4) A less pronounced negative FB was associated with worsening kidney function. (5) The use of noradrenaline was linked to an increased risk of kidney function deterioration following furosemide administration.

Fluid accumulation is common in critically ill patients—not only due to resuscitation efforts but also from maintenance fluids, nutrition, and medications. Although fluid removal is central to the de-resuscitation phase, research remains limited regarding its optimal timing, goals, and methods [16]. Furosemide is commonly administered to actively promote fluid removal during de-resuscitation; however, the predictability of achieving a predetermined negative FB is challenging, and the efficacy of diuretic use varies significantly among patients.

MAP’s strong association with diuretic response may reflect its influence on kidney perfusion. During post-stabilization and early de-resuscitation, vascular tone improves and extravascular fluid reenters circulation, increasing stressed volume, venous return, and cardiac output [16,21]. This rise in MAP enhances renal blood flow and diuretic response [22]. Furthermore, with over half of the study population affected by hypertension, it is plausible that some patients exhibited a rightward shift in the kidney’s autoregulatory curve. This shift could make these individuals more responsive to diuretics only at higher MAP levels. This MAP–kidney interaction may represent an important consideration in the management of fluid overload in critically ill patients.

Parameters such as noradrenaline support, SOFA score, BUN and serum creatinine levels may serve as indicators of illness severity and kidney performance, potentially signaling a persistent “ebb phase” or organ damage from initial disease insults, and possibly a “third hit” from global increased permeability syndrome [16]. Within the low-to-moderate MAP range, impaired kidney perfusion and function significantly limited fluid removal. At higher MAP levels, elevated SOFA scores remained strongly and inversely associated with achieving a negative FB, suggesting persistent organ dysfunction despite stable hemodynamics. This may signal difficulty transitioning to the “flow phase” and a reduced response to de-resuscitation efforts. Although the SOFA score is primarily intended to quantify the progression of organ dysfunction rather than define specific shock phases, it appears to share a clinical association with fluid status during the ICU stay. Cordemans et al. observed that SOFA scores were significantly lower in patients who achieved conservative late fluid management, defined as maintaining a neutral-to-negative fluid balance for at least two consecutive days within the first week of intensive care [15].

Impaired kidney function and noradrenaline support were associated with an attenuated negative FB following initial furosemide administration, highlighting the impact of combined renal dysfunction and hemodynamic instability on fluid removal. It remains unclear whether noradrenaline directly contributes to reduced diuretic responsiveness or serves primarily as a surrogate marker for illness severity. While prospective studies are warranted to delineate the impact of vasopressors on fluid balance across varying MAP levels and disease stages, these findings suggest that achieving negative fluid balance in patients requiring noradrenaline may necessitate highly individualized strategies, including the timely consideration of kidney replacement therapy.

Albumin levels have been associated with furosemide efficacy [23,24,25] and hypoalbuminemia, often due to increased capillary permeability, is linked to poorer outcomes and higher mortality [15,23,26]. In this study, however, plasma albumin levels showed only a modest association with the likelihood of achieving negative FB. This finding suggests a more nuanced relationship between albumin levels, colloid osmotic pressure, and FB regulation [22]. One possible explanation is that the mean albumin levels in our cohort were significantly higher than those reported in studies linking low albumin with endothelial surface damage, which may impact fluid dynamics [21,27].

While statistically significant changes in creatinine and BUN levels were observed following the initiation of furosemide, particularly among patients achieving a less negative fluid balance, the absolute magnitude of these fluctuations was clinically modest and should be interpreted with caution. Similarly, variations in serum sodium levels were minor and lacked clinical relevance despite reaching statistical significance.

Numerous studies have linked fluid overload with increased morbidity and mortality in critically ill patients [15,28]. Conversely, restrictive fluid management strategies are linked to reduced mortality and improved clinical outcomes, including more ventilator-free days and shorter ICU stays [4,10,15]. Following the initial acute phase of illness and subsequent stabilization, an efficient de-resuscitative phase is essential to mitigate a “third hit,” characterized by continued organ stress due to persistent fluid overload. This involves both limiting intake and actively removing excess fluid accumulated during earlier care [15,16]. However, further research is necessary to identify the optimal timing, methods, and specific goals for achieving a safe and effective negative FB during de-resuscitation. This study provides a deeper understanding of achieving a pre-determined negative FB and the factors influencing its attainment.

To our knowledge, this is the first large-scale study utilizing comprehensive clinical data to examine the associations between clinical variables and furosemide response during the de-resuscitation phase. While MAP may reflect a patient’s underlying readiness for de-resuscitation rather than acting as a direct driver of diuretic efficacy, it nonetheless serves as a valuable clinical signal. These findings offer important insights into real-world fluid management practices during the active fluid removal stage of critical care.

This study employed two complementary statistical approaches: CHAID decision tree analysis to explore associations with the initial furosemide dose, and mixed model analysis to evaluate repeated measurements over time. CHAID enabled identification of complex interactions among categorical and continuous variables, while mixed models accounted for intra-patient variability and missing data. This approach provided a framework to quantify the influence of clinical factors on daily FB, supporting personalized treatment strategies.

This study has several limitations. First, it was conducted at a single center, which may limit the generalizability of the findings. Second, prior diuretic use was inconsistently documented, potentially affecting the observed responses. Third, the absence of a standardized furosemide administration protocol introduced variability in clinical practice, which may have influenced outcomes. Fourth, although a broad range of clinical variables was included, the lack of granular data, specifically regarding pre-existing chronic kidney disease, baseline creatinine levels, precise patient weights, and exposure to nephrotoxic medications, remains a limitation. The inclusion of these factors might have provided deeper insights into the observed variability in diuretic response and would have allowed for the formal staging of acute kidney injury according to consensus criteria, such as the KDIGO guidelines. While such data were not consistently available within this retrospective cohort, they represent critical areas for future prospective research aimed at refining individualized fluid management strategies. Finally, the MAP thresholds identified by the CHAID decision tree are inherently data-driven and were derived without internal or external validation; accordingly, they should be interpreted as exploratory and hypothesis-generating rather than as prescriptive clinical targets and should not be extrapolated to other populations or clinical protocols without prospective validation.

## 5. Conclusions

In this cohort, MAP was strongly and continuously associated with the achievement of negative fluid balance during de-resuscitation. When evaluated alongside secondary parameters, these clinical correlations may provide a clearer picture of diuretic responsiveness. While these findings support the adoption of individualized de-resuscitation strategies, prospective studies are warranted to further characterize these associations and determine their clinical utility in real-time practice.

## Figures and Tables

**Table 1:** Baseline characteristics of the cohort (1764 patients, 6632 patient’s days in the ICU) on the morning prior to the initiation of furosemide therapy.

Value		
Age (years); [mean, Std]	61.73	17.69
Male [N, %]	1030	58.4%
ICU Length of stay *^a^*^,*b*^ (Days); [median, IQR]	6.9	[3.3, 12.9]
SOFA *^a^* [median, IQR]	5	[3, 7]
Daily Fluid balance (mL/24 h) ^*c*^ [mean, Std]	−956	1232
Daily Urine (mL/24 h) ^*c*^ [mean, Std]	2222	1184
Daily Drain and naso-gastric output (mL/24 h) *^c^* [mean, Std]	143	313
Daily Fluids intake (mL/24 h) ^*c*^ [mean, Std]	585	535
Daily Enteral Nutrition (mL/24 h) ^*c*^ [mean, Std]	781	682
Avg. daily HR *^a^* (1/min); [mean, Std]	85.3	14.8
Avg. daily MAP *^a^* (mmHg); [mean, Std]	83.1	12.8
Avg. peak Temperature (°C); [mean, Std]	37.4	0.6
Creatinine *^b^* (mg/dL); [mean, Std]	1.2	1.1
Albumin *^b^* (g/L); [mean, Std]	28.5	4.8
Sodium *^b^* (mmol/L); [mean, Std]	141.9	5.4
Blood Urea Nitrogen *^b^* (mg/dL); [mean, Std]	37	25.1
Hemoglobin *^b^* (gr/dL); [mean, Std]	9.4	1.8
Arterial blood gas-Base Excess (mmol/L); [mean, Std]	3.8	5.8
Arterial blood gas-HCO_3_ (mmol/L); [mean, Std]	29	6
Co-morbidities:		
Hypertension [N, %]	921	52.2%
Diabetes [N, %]	709	40.2%
Heart failure [N, %]	100	5.7%
Ischemic heart disease [N, %]	251	14.2%
Cirrhosis [N, %]	42	2.4%
Prior cardiac surgery [N, %]	20	1.1%
Post operative admission [N, %]	256	14.5%
Trauma [N, %]	90	5.1%
Sepsis [N, %]	372	21.1%

*^a^* ICU—Intensive Care Unit; SOFA—Sequential Organ Failure Assessment; HR—Heart Rate; MAP—Mean Arterial Pressure; *^b^* Value recorded prior to the first furosemide administration. *^c^* Daily measurement calculated from the morning preceding furosemide administration to the following morning.

**Table 2:** Mixed Model Analysis of Clinical Variables Associated with Fluid Balance.

Variable	Balance Change per 1 Variable Unit (mL/24 h)	Std. Error	*p*-Value	95% CI (Lower, Upper)
Avg. daily MAP (mmHg)	−23.3	1.476	<0.001	(−26.19, −20.40)
Noradrenaline Support *^d^*	+327.96	51.264	<0.001	(227.46, 428.45)
Furosemide Dose (mg)	−3.42	0.304	<0.001	(−4.01, −2.82)
Creatinine (mg/dL)	+173.53	23.297	<0.001	(127.85, 219.21)
Albumin (g/dL)	+14.71	4.406	<0.001	(6.07, 23.35)
Age (years)	+9.65	1.425	<0.001	(6.85, 12.44)
Daily SOFA score	+28.24	7.822	<0.001	(12.91, 43.58)
Length of Stay (days)	+4.06	1.457	0.005	(1.20, 6.91)
Bicarbonate (mmol/L)	−13.16	3.919	<0.001	(−20.85, −5.48)
Peak Temperature (°C)	−62.67	30.89	0.043	(−123.23, −2.11)
Sodium Levels (mmol/L)	−3.13	3.913	0.425	(−10.80, 4.55)
Daily Average Heart Rate	+0.74	1.372	0.59	(−1.95, 3.43)
Hypertension	+24.54	53.937	0.649	(−81.26, 130.33)
Ischemic Heart Disease	−41.12	69.437	0.554	(−177.31, 95.08)
Cirrhosis	−62.22	177.971	0.727	(−411.26, 286.81)
Congestive Heart Failure	+194.73	111.588	0.081	(−24.12, 413.58)
Metastatic Cancer	−289.81	222.592	0.193	(−726.40, 146.78)
Diabetes	−5.26	53.442	0.922	(−110.09, 99.57)

*^d^*—Noradrenaline support regardless of dose.

## Data Availability

The data presented in this study are available on request from the corresponding author. (please specify the reason for restriction, e.g., the data are not publicly available due to privacy or ethical restrictions).

## Abbreviations

The following abbreviations are used in this manuscript:

MAP	Mean arterial pressure
ICU	Intensive care unit
FB	Fluid balance
SOFA	Sequential organ failure assessment
BUN	Blood urea nitrogen
CHAID	Chi-squared Automatic Interaction Detector
